# FONZIE: An optimized pipeline for minisatellite marker discovery and primer design from large sequence data sets

**DOI:** 10.1186/1756-0500-3-322

**Published:** 2010-11-29

**Authors:** Pascal Bally, Jonathan Grandaubert, Thierry Rouxel, Marie-Hélène Balesdent

**Affiliations:** 1 Institut National de la Recherche Agronomique, UMR 1290 BIOGER, BP 01, Avenue Lucien Brétignières, 78850 Thiverval-Grignon, France

## Abstract

**Background:**

Micro-and minisatellites are among the most powerful genetic markers known to date. They have been used as tools for a large number of applications ranging from gene mapping to phylogenetic studies and isolate typing. However, identifying micro-and minisatellite markers on large sequence data sets is often a laborious process.

**Results:**

FONZIE was designed to successively 1) perform a search for markers via the external software Tandem Repeat Finder, 2) exclude user-defined specific genomic regions, 3) screen for the size and the percent matches of each relevant marker found by Tandem Repeat Finder, 4) evaluate marker specificity (i.e., occurrence of the marker as a single copy in the genome) using BLAST2.0, 5) design minisatellite primer pairs via the external software Primer3, and 6) check the specificity of each final PCR product by BLAST. A final file returns to users all the results required to amplify markers. A biological validation of the approach was performed using the whole genome sequence of the phytopathogenic fungus *Leptosphaeria maculans*, showing that more than 90% of the minisatellite primer pairs generated by the pipeline amplified a PCR product, 44.8% of which showed agarose-gel resolvable polymorphism between isolates. Segregation analyses confirmed that the polymorphic minisatellites corresponded to single-locus markers.

**Conclusion:**

FONZIE is a stand-alone and user-friendly application developed to minimize tedious manual operations, reduce errors, and speed up the search for efficient minisatellite and microsatellite markers departing from whole-genome sequence data. This pipeline facilitates the integration of data and provides a set of specific primer sequences for PCR amplification of single-locus markers. FONZIE is freely downloadable at: http://www.versailles-grignon.inra.fr/bioger/equipes/leptosphaeria_maculans/outils_d_analyses/fonzie

## Background

Satellite sequences are abundantly interspersed in the genome of almost all eukaryotic species studied [1] and have been analyzed extensively in animals [2-4], plants [5], and more recently in fungi [6].

Two major classes of satellites are usually defined: minisatellites (MS) and microsatellites (μS). μS are repetitive sequences of mostly 2 to 4 nucleotides with a widespread occurrence in multicellular organisms, whereas minisatellites are usually defined as the repetition in tandem of a short, 6 to 100 bp motif, spanning 100 bp to several kilobases [7].

μS analysis requires sophisticated separation and visualization apparatus, due to the small size of the individual repeat units. In contrast, and because of the larger size of their individual core motif, MS can be separated and visualized by conventional agarose gel electrophoresis [6]. Using a panel of single-locus minisatellite markers, isolate-specific DNA fingerprint can be produced in a PCR-based assay [8,9]. MS also provided highly polymorphic markers for linkage studies [10] making them informative genetic markers.

Genome mapping is still the primary tool for genome knowledge in a series of model organisms. Due to their small genome size and laboratory tractability, fungal models such as the ascomycete yeast *Saccharomyces cerevisiae*, and the filamentous ascomycete *Neurospora crassa*, have been pioneers in the process of building genetic maps [11]. In this respect, the Dothideomycete *Leptosphaeria maculans *causing stem canker of oilseed rape (*Brassica napus*), is amenable to genetics in the lab and is thus an adequate and complete model for genome-wide-based functional studies of pathogenicity, and for identifying signalling and regulation processes responsible for shifts in lifestyle [12]. Although preliminary genetic maps have been developed for *L. maculans *[13-15], it is difficult to integrate these maps as the markers (RAPD and AFLP) used to generate them are not readily transferable [16]. Based on the analysis of a set of around 800 BAC-end sequence data, MS were found to be powerful for genetic mapping or population genetic studies in *L. maculans *[6]. The availability of the *L. maculans *genome now enables the generation of a large quantity of such markers, which could allow the saturation of the genetic map and improve population genetic studies and pathogenicity gene discovery [12].

Despite the availability of whole genome sequences, *in silico *MS identification and primer design is still a laborious process, requiring the use of different softwares and numerous steps of marker validation, which take time and often generate errors. Most of the currently available software or pipeline solutions are focused on the identification of μS [17-19]. Moreover, they are usually platform dependent [20], or may need several tedious pre-processing steps [19,21]. In order to avoid these constrains and to reduce time, FONZIE has been developed to automate and facilitate the design of PCR primers from large sets of sequences, that will amplify single locus MS and their flanking sequences, with the possibility of excluding some specific regions of the genome from the MS design process. This pipeline integrates various external tools such as BLAST [22], Tandem Repeat Finder (TRF) [23], and Primer3 [24]. FONZIE is able to successively perform TRF on a Fasta or Multifasta formatted sequence file, search for markers specifically located in user-defined sequence subset, as exemplified here with GC-equilibrated isochores of *L. maculans*, eliminate markers which do not satisfy user-defined criteria, eliminate markers which are not single-copy, and finally design minisatellite primer pairs and check the specificity of the PCR product. The primary targeted public of FONZIE is biologists unfamiliar with complex bioinformatics solutions. Therefore it does not require a computer science background and can run on personal computers, yielding results within an hour or less for most queries.

## Implementation

A comprehensive "readme" page includes details on how to configure and run the pipeline, and how to search and display FONZIE results.

Before launching FONZIE, users must have installed the Python programming language on the computer. In a second step, users must create a database of sequences (ideally, the whole-genome sequence of the investigated organism) in order to perform BLAST during the execution of the pipeline. In our project, the database used was the pilot genome of *L. maculans *(45.12 Mb, 76 Super-Contigs, Table 1) as a Multifasta file.

**Table 1:** FONZIE results when performed on different fungal or oomycetes whole genomes.

				Nb of markers identified^c^			Nb of amplification products (AP) and primers designed^d^				
					
Organism	Genome Size (Mb)	Nb of contigs or super contigs^a^	Execution Time^b^	Total	Single copy	Multiple copies	Single copy	Multiple copies	No primers	No BLAST results	% single-copy AP
*Aspergillus niger*	34.85	24	2 min 53 sec	637	533	104	600	18	17	2	94.19
*Stagonospora nodorum*	37.21	108	4 min 41 sec	1288	978	310	959	154	169	6	74.46
*Pyrenophora tritici repentis*	37.84	47	5 min 12 sec	1141	878	263	935	195	8	3	81.94
*Botrytis cinerea*	42.66	588	12 min 03 sec	3648	2719	929	3339	118	176	15	91.53
*Leptosphaeria maculans*	45.12	76	23 min 36 sec	2606	1799	807	2405	146	49	6	92.29
*Laccaria bicolor*	64.88	665	45 min 39 sec	5393	1516	3877	1640	3409	338	6	30.41
*Phytophthora infestans*	228.54	4921	3 h 21 min 37 sec	7514	1855	5659	1718	5239	553	4	22.86

^a ^Number of sequences in the Multifasta file

^b ^Machine used for this test: Laptop Intel Core 2 Duo, 2.4 GHz and 3Go RAM

^c ^FONZIE results after step d of the workflow (Figure 1), using the TRF default parameters (match = 2, indel = 7, mismatch = 7, pi = 10, pm = 80, minscore = 50, maxperiod = 500) and screen parameters for core motif size >3, % identity between motifs = 90%, BLAST cut-off value = 1e-10)

^d ^Final FONZIE results after steps e (Primer pair design) and f (checking for the specificity of the amplification product) of the workflow shown in Figure 1, using a BLAST cut-off value of 1e-40

### Pipeline Components

**Figure 1:**
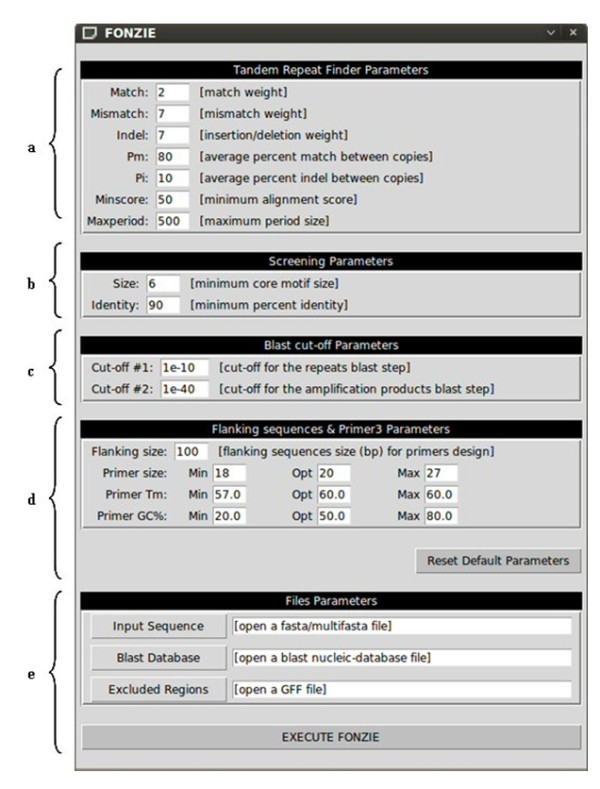
**The graphical user interface of FONZIE**. The graphical user interface is composed of five major sections. **a**. TRF parameters: All the modifiable parameters of TRF are located in this section. Users can modify each of them separately. **b**. Screening parameters. Users can modify the core motif size and the percent match between core motifs **c**. BLAST cut-off parameters: Users can modify the two E-value cut-off used during the screening for marker specificity and the virtual PCR steps **d**. Flanking sequences size and Primer3 parameters. **e**. The file field: In this section, users can open the Fasta- or Multifasta formatted sequence file, the BLAST database file, and the optional gff format file which contains the excluded regions.

FONZIE consists of three major components: a set of pipelined programs, a BLAST database and a graphical user interface (Figure 1). The FONZIE pipeline consists of Python modules allowing the execution of different external software programs.

The BLAST database was created with FORMATDB, a BLAST [22] database-related tool. The graphical user interface was implemented with a Python graphic standard library named Tkinter. FONZIE was developed under both Linux Ubuntu 7.10 and Windows XP operating systems.

**Figure 2:**
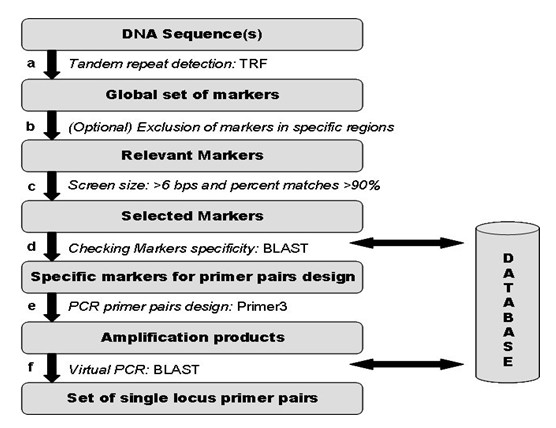
**The FONZIE workflow**: The FONZIE workflow consists of 6 steps. a Execution of TRF on a Fasta sequence or a Multifasta file, b Optional: exclusion of some user-defined specific regions, c Screening by core motif size and percent match parameters, d Screening for marker specificity with BLAST (1e-10 by default) against a database, e Primer design step using PRIMER3, f Virtual PCR by making a BLAST (1e-40 by default) against the database to check the specificity of the PCR product (single copy locus).

The automated process consists of six steps (Figure 2):

#### Tandem Repeat Finder

FONZIE accepts nucleotidic sequence files in Fasta or Multifasta format and uses the Tandem Repeat Finder software [23] in order to find tandemly repeated elements (minisatellites and microsatellites) (Figure 2 step a). TRF parameters can be modified via the graphical user interface (Figure 1).

#### Exclusion of markers in specific regions

Isochores have been described for many eukaryotic organisms such as plants and mammals [25,26]. Isochores are very long stretches of DNA that are homogeneous in base composition and are compositionally correlated with the coding sequences that they embed. In the *L. maculans *genome this structure is particularly clear, with alternation of GC-equilibrated coding regions with no or few transposable elements (TE) and AT-rich regions mainly composed of degenerated and truncated TE and encompassing very few ORFs [13,27]. Due to these particularities, most micro-or minisatellites identified in *L. maculans *AT isochores are not single locus markers and have to be excluded from the search for single locus markers. This can be easily extended to all repeat-rich genomes such as that of the Oomycete *Phytophthora infestans *containing 74% of repeats [28].

In order to exclude such specific genomic regions from the search for markers, FONZIE compares the location of the tandem repeats with that of the AT-rich isochores (or whichever other user-defined sequences). Tandem repeats fully or partially overlapping the AT-rich isochores (or other user-defined sequences) are thus automatically excluded from the analysis (Figure 2 step b). The AT-rich isochores or other user-defined sequences to be excluded must be specified in a GFF format file. If no isochore regions, or any other regions to be excluded from the search for MS, are specified, FONZIE analyses all the tandem repeats found by TRF.

#### Length and percent match screening

Markers are then screened for the size of the core motif (more than 6 bp by default), and the percent matches between core motifs (i.e., the percentage of matched bases between tandem repeats of the core motif within the MS, more than 90% by default). A minimum of 90% identity between core motifs was selected here because previous experiments showed that it was a characteristic of minisatellites showing actual sequence length polymorphism in *L. maculans *[[6]; unpublished data]. The output of this step is a Fasta formatted sequence file for each selected marker (Figure 2 step c).

#### Screening for specificity of the markers

This step verifies the specificity of the selected MS (Figure 2 step d). Each MS is analysed for sequence similarity using BLAST [22] against a BLAST database, created by users, with a default E-value cut-off set at 1e-10. This value can be modified via the graphical user interface. Typically, the specificity is evaluated against the whole genome sequence if available.

Using the output of the BLAST, FONZIE classifies MS into 4 categories, defined by both the number of sequences in the BLAST database matching the query sequence (i.e. with BLAST E-value < 1e-10) and the E-value obtained for the query sequence compared to that obtained for other matching sequences, as follows:

1) MS for which the BLAST results exhibit a unique match in the database, corresponding to the query sequence, hereafter referred to as "UNIQUE COPY".

2) MS for which the BLAST results exhibit several matches on several sequences, with a best hit on the query sequence, hereafter referred to as "MULTIPLE COPIES".

3) MS for which the BLAST results exhibit several matches on several sequences, with a best hit on other sequence than the query sequence, hereafter referred to as "MULTIPLE COPIES (BEST HIT ON SEQUENCE X)". X represents the sequence id. with the best alignment score. The repetition of the motifs among the whole genome can explain such results.

4) (Rare) MS for which the BLAST results exhibit no match on any sequences with the default cut-off of E-value at 1e-10, hereafter referred to as "NO BLAST RESULTS" (0.2% in the case of *L. maculans*, Table 1). The high default threshold parameter of the E-value (1e-10), the low complexity of the motifs, or the small size of the MS sequence can explain such results.

"MULTIPLE COPIES (BEST HIT ON SEQUENCE X)" and "NO BLAST RESULTS" are automatically excluded for the next steps of the analysis.

#### Primer design

In order to design primer pairs flanking the MS sequences, FONZIE extracts 100 bps by default on each side of the query sequence whenever possible (or whichever other user-defined length, modified via the graphical user interface, Figure 1). Otherwise, FONZIE extracts the maximum number of nucleotides available upstream and downstream of the MS sequence.

FONZIE then uses Primer3 [24] which analyses the flanking DNA sequences in order to define suitable forward and reverse PCR primers, designed with the standard set of constraints of Primer3 (Figure 2 step e): optimal primer size at 20 bases pairs, optimal Tm at 60°C, and optimal primer GC content at 50%. These parameters can also be modified by users via the graphical user interface (Figure 1). FONZIE then calculates the final product size range as the size of the MS (= the size of the core motif × the number of repeats as defined by TRF) + 2×, or 10×, the minimal primer size (chosen by users), for the minimal and the maximal size, respectively. The first primer pair returned by Primer3 is displayed by FONZIE. If no primer pairs are found with Primer3, the result is displayed as "NO DESIGNED PRIMER" in the final text file.

#### Virtual PCR

The virtual PCR step validates the specificity of amplification products identified in step e (Figure 2 step f). Each amplification product is analysed for sequence similarity using BLAST against all the sequences of the BLAST database. The E-value cut-off is fixed at 1e-40 by default and may be modified by the users. The same type of analysis is implemented in the SAT software [19], but SAT only verifies the specificity of the PCR primers, whereas FONZIE analyses the specificity of the whole amplification product.

Using the outputs of the virtual PCR step, FONZIE classifies sequences in 4 categories according to the specificity of amplification products. These categories are exactly the same as for the markers' specificity screen.

### Running the pipeline

Users have to provide an input sequence (Fasta or Multifasta format), and a GFF format file if they want to exclude specific regions from the analysis.

#### Setting parameters and pipeline execution

TRF default options are as follows: alignment parameters of 2,7,7, which correspond to the weight attributed to each match, mismatch and indel, respectively, between core motifs of the MS; minimum alignment score of 50; maximum period size of 500, pM (i.e., the average percent identity between the copies of a pattern) of 80; pI (i.e., the average percent of insertions and deletions between the copies of a pattern) of 10 (Figure 1) [23]. Users can modify through the graphical interface all TRF parameters, but also the screening parameters (i.e., the minimum identity between core motif fixed by default at 90%, and the size of the motif, >6 bps by default) as well as the two BLAST cut-off parameters (E-values cut-off of 1e-10 and 1e-40 by default) (Figure 1).

#### Visualization of results

At the end of the process, users have access to all the files and directories created at each step of the pipeline. A final tab-delimited text file summarizes, for all numbered satellites found by FONZIE, the name of each satellite automatically attributed by the pipeline, the core motif of each satellite, the number of repetitions of the core motif, the BLAST results of specificity of the satellite and of the amplification product (i.e. if the marker and the amplification product are found as a unique copy or not), the sequences of the forward and reverse primers and the start and the end location of the amplification product on the input sequence (Table 2).

**Table 2:** Example of the FONZIE final result table, run on Supercontig 16 of the *Leptosphaeria maculans *genome.

MARKER_ID	**MOTIF**^**a**^	**REPETITION**^**b**^	**MARKER_STATUS_ON_GENOMELEPTOV2**^**c**^	**LEFT_PRIMER**^**d**^	**RIGHT_PRIMER**^**e**^	**START_AMPLIF_PRODUCT_ON_SUPERCONTIG_16**^**f**^	**END_AMPLIF_ PRODUCT_ON_SUPERCONTIG_16**^**g**^	**STATUS_AMPLIF_ PRODUCT_ON_GENOMELEPTOV2**^**h**^
min_supercontig_16_10	ATAAAAGTAAACTACTACTTTA	2.0	MULTIPLE_COPIES	GCATAAAGCTAATCTTCTCTACCCC	GTATAAACTGCCCTTGTGTATACCT	100841	101019	MULTIPLE_COPIES

min_supercontig_16_11	GGATCATCAAGGA	17.3	UNIQUE_COPY	CGTTTTGGCTTTGTTGTTGA	ACTATGAGCCAGGTGAACCG	111896	112241	UNIQUE_COPY

min_supercontig_16_12	CGCTCTCTCTCTCTCTCTTTCTCTCT	4.3	MULTIPLE_COPIES	CGCCAACAAGACTACCCATC	GAAGCGGTGGCAGTTTTTAG	112524	112812	UNIQUE_COPY

min_supercontig_16_13	CCATGT	5.8	UNIQUE_COPY	ACCTCCCGAGGAAAAGTGAC	CTTGTGTGGTCTGGTTGCAG	134392	134595	UNIQUE_COPY

min_supercontig_16_14	GAGAGAGAGAGAGAGAGA	7.4	MULTIPLE_COPIES	TGACTCGGCGTCTACCCTAC	AGCCAGCCAGCCAGTACTAA	136186	136390	UNIQUE_COPY

min_supercontig_16_15	AAGCAGAAGGCTATTGAGTCGCCAGAGACAAGTCCACAGTCC	2.1	UNIQUE_COPY	AAGTGGCTGGACCTAGCAGA	ACATCGGCGACACGTTTAGT	142179	142347	UNIQUE_COPY

min_supercontig_16_16	GTGTGG	11.2	MULTIPLE_COPIES	TGTGGATGATAGGATGGGGT	GTGACAAGCACATGATTCGC	156524	156707	UNIQUE_COPY

Only a few markers generated by FONZIE are displayed in the table.

^a ^consensus sequence of the core motif of the minisatellite (MS)

^b ^number of repeats of the core motif

^c ^results of the first BLAST step on the BLAST database: UNIQUE_COPY, the unique sequence matching the MS (query) is the query sequence; MULTIPLE_COPIES, more than one sequence of the BLAST database match the query sequence and the best hit is obtained for the query sequence (E-value cut-off e-10)

^d ^and ^e^, sequences of the left and right primers, respectively, generated by Primer3 to amplify the minisatellite locus and flanking sequences

^f ^and ^g^, location (in base pairs) of the primers along Super-Contig 16 sequence.

^h^, results of the second BLAST step, where the amplification product is blasted on the BLAST database: UNIQUE_COPY, the unique sequence matching the PCR product (query) is the query sequence; MULTIPLE_COPIES, more than one sequence of the BLAST database match the query sequence and the best hit is obtained for the query sequence (E value cut-off e-40).

#### Sequence files and directory

FONZIE allows traceability of each step of the pipeline by generating a directory and associated files. FONZIE displays a DAT file created by TRF which contains the information of all tandem repeats detected on the input sequence. A text file shows the different consensus sequences of each marker, its position on the input sequence as well as period size, copy number, consensus size, percent matches, percent indels, the score and the status of each marker.

**Figure 3:**
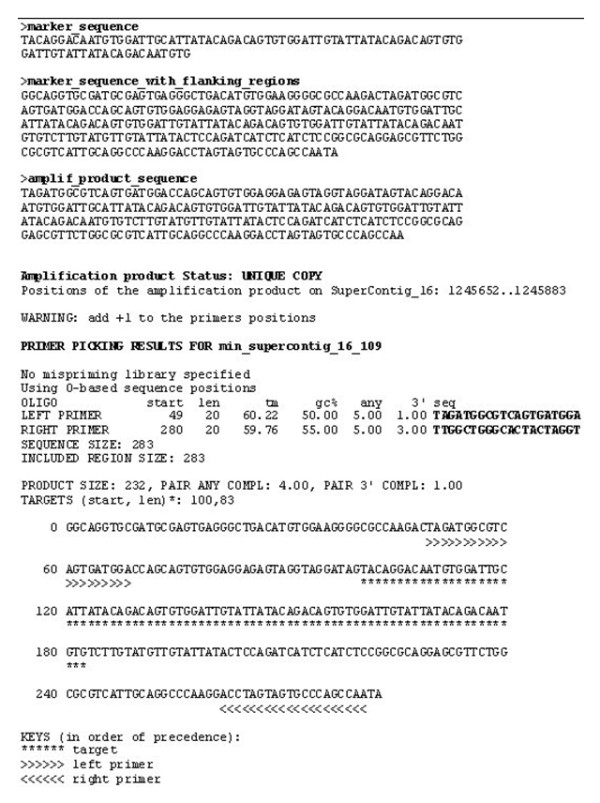
**Example of one file from "MARKERS_RECAP" directory generated by FONZIE**. From top to bottom: the marker sequence, the marker sequence with flanking regions, the amplification product sequence, and the primer3 output

A directory named "MARKERS_SEQUENCES" is created at the end of the first screening step which contains all the markers found by FONZIE, in Fasta format. Another directory named "AMPLIF_PRODUCTS_SEQUENCES", contains the Fasta files for the sequence of each amplification product. A last directory named "MARKERS_RECAP" contains, for each MS, a text file summarizing the marker sequence, the marker sequence with flanking regions, the amplification product sequence, the amplification product status, the positions of the amplification product on the query sequence, and the output of Primer3 indicating the characteristics of the primers and their location in relation to the MS (Figure 3).

## Results and discussion

### Performance

FONZIE was firstly assessed on the whole *L. maculans *genome. The 45 Mbs were analysed in 23 minutes (Table 1). FONZIE found 2606 minisatellites, with 2405 amplification products occurring as unique copies, 146 in multiple copies. Six amplification products had no BLAST results, and 49 markers were identified for which no primers could be designed. 92% of the markers identified by FONZIE, following exclusion of repeat-rich AT-rich isochores thus corresponded to putatively unique sequences in the genome and could be further used for analysis of polymorphism in isolates and genetic mapping and/or population genetics studies.

In a second step, FONZIE was used to mine 6 other fungal and Oomycete genomes. These genomes, sized from 34 Mb to 228 Mb (Table 1), range from compact, repeat poor genomes (*Aspergillus niger*, *Stagonospora nodorum*, *Pyrenophora tritici repentis *and *Botrytis cinerea*) to repeat rich genomes of the basidiomycete *Laccaria bicolor *[29] and the Oomycete *P. infestans *[28]. MS were found using the same parameters as for *L. maculans*, but without defining user-specified genomic regions to be excluded from the analysis. In all cases, numerous MS could be identified and extracted in a very limited time interval, i.e. between 2 minutes to cover the *A. niger *whole genome to 3 h21 min to scan the *P. infestans *genome sized 228 Mb over 4921 contigs (Table 1). This analysis suggests some genomes are extremely rich in MS (e.g., the *B. cinerea *genome; Table 1). It mainly indicates that an average of 70-83% single-copy markers are identified whenever the genomes investigated are poor in repeats or when repeat-rich regions are excluded from the analysis, as done here *for L. maculans *(Table 1). In contrast, only 23-30% of markers were found to be single-copy markers in the genomes of the repeat-rich fungi and Oomycete *L. bicolor *and *P. infestans *if analysing the whole genomes with FONZIE, without user-defined exclusion of repeat-rich sequences.

### Biological validation

For validation of the proposed approach, primer pairs for 517 putative single-copy MS markers (i.e. with core motif ≥6), scattered along 17 *L. maculans *supercontigs (with AT-rich isochores as excluded regions), were PCR-assayed on a set of 9 *L. maculans *natural isolates. More than 90% of the primer pairs (475) amplified a PCR product, with size compliant with the expected MS size. This yield is very similar to that of the currently available tools such as STAMP, yielding 83.33 to 91.67% of successful amplifications, depending on the species [30]. Among these PCR-amplified sequences, 44.8% (213 primer pairs) showed resolvable size polymorphism between at least 2 of the 9 tested *L. maculans *isolates. Finally, 24.8% of the 517 MS (i.e. more than 55% of the polymorphic MS markers) were found to be polymorphic between isolates 'a.2' and 'H5', the two parental isolates of the *L. maculans *genetic map [13]. Progeny analysis confirmed that all these markers segregated as single loci, i.e. with a 50:50 ratio, as expected for this haploid organism.

In previous experiments, MS markers were searched for in *L. maculans *by using the TRF application alone [6], with a yield of only 13% of polymorphic minisatellites out of 30 primer pairs tested, even though a wider range of isolates (21 isolates instead of 9 in the present study) was used for the polymorphism screen. Only 10% of the primer pairs from this preliminary study were found to be polymorphic between isolates 'a.2' and 'H5', compared to 24.8% in the present work. These results not only validate the efficiency of the pipeline but also confirm the potential of MS as genetic markers in *L. maculans*, since it can be extrapolated both from Table 1 and these results that more than 530 MS could be mapped to the reference *L. maculans *map.

## Conclusion

FONZIE is a user-friendly application, optimised for MS marker identification and specific PCR primer design. It brings together external software like Tandem Repeat Finder, Primer3 and BLAST, to perform them in one run for PCR primer pair design. FONZIE is time-saving, reduces errors that might be introduced by analysing sequence by hand and is easy to use. Users only need to upload their sequences, a reference database, an optional file containing excluded genomic regions, and select appropriate parameters. Moreover, FONZIE can cover whole genomes in one run. This tool is generic enough to be used on the DNA of any organism and is particularly well adapted to whole genome sequence from which specific regions (AT-isochores for examples) need to be excluded.

The main innovation of the pipeline is to integrate two successive steps of BLAST to check the specificity and uniqueness of the MS loci identified by TRF. The usefulness of the pipeline has been demonstrated here for the fungus *L. maculans*, since 100% of the polymorphic markers identified were single locus, and the yield of identification of polymorphic markers has been more than doubled compared to manual search for MS without BLAST steps.

## Availability and requirements

• Project name: FONZIE

• Operating system: UNIX/LINUX, Windows XP

• Programming language: Python

• Licence: GNU GPL, free for academic and non-academic users

• Any restrictions to use by non-academic: none

## Competing interests

The authors declare that they have no competing interests.

## Authors' contributions

PB and JG designed the application, JG programmed the application and supervised the implementation of the computational part of this work, PB and JG tested and evaluated the application, TR contributed to validation on fungal genomes and edited the manuscript, PB and MHB performed the biological validation of the markers on *L. maculans*, PB wrote the manuscript, MHB initiated and coordinated the work and helped to draft the manuscript. All authors read and approved the final manuscript.
